# Solid-Gas Phase Photo-Catalytic Behaviour of Rutile and TiO_n_ (1 < n < 2) Sub-Oxide Phases for Self-Cleaning Applications

**DOI:** 10.3390/ma12010170

**Published:** 2019-01-07

**Authors:** Manuel Nuño, Vaia Adamaki, David M. Tobaldi, Maria J. Hortigüela Gallo, Gonzalo Otero-Irurueta, Chris R. Bowen, Richard J. Ball

**Affiliations:** 1 BRE Centre for Innovative Construction Materials (CICM), Department of Architecture and Civil Engineering, University of Bath, Claverton Down, Bath BA2 7AY, UK; manuelnunotutor@hotmail.com; 2 Department of Mechanical Engineering, University of Bath, Claverton Down, Bath BA2 7AY, UK; V.Adamaki@bath.ac.uk (V.A.); c.r.bowen@bath.ac.uk (C.R.B.); 3 Department of Materials and Ceramic Engineering/CICECO—Aveiro Institute of Materials, Campus Universitário de Santiago, University of Aveiro, 3810-193 Aveiro, Portugal; david.tobaldi@ua.pt; 4 Center for Mechanical Technology and Automation—TEMA, Department of Mechanical Engineering, Campus Universitário de Santiago, University of Aveiro, 3810-193 Aveiro, Portugal; mhortiguela@ua.pt (M.J.H.G.); otero.gonzalo@ua.pt (G.O.-I.)

**Keywords:** photocatalysis, TiO_2_, sub-oxide

## Abstract

The solid-gas phase photo-catalytic activities of rutile TiO_2_ and TiO_n_ (1 < n < 2) sub-oxide phases have been evaluated. Varying concentrations of Ti^3+^ defects were introduced into the rutile polymorph of titanium dioxide through carbo-thermal reduction at temperatures ranging from 350 °C to 1300 °C. The resulting sub-oxides formed were characterized by X-ray diffraction, X-ray photoelectron spectroscopy, scanning electron microscopy, impedance spectroscopy and UV-visible diffuse reflectance spectroscopy. The presence of Ti^3+^ in rutile exposed to high reduction temperatures was confirmed by X-ray diffraction. In addition, a Ti^3+^-Ti^4+^ system was demonstrated to enhance the photo-catalytic properties of rutile for the degradation of the air pollutants NO_2_ and CO_2_ under UV irradiation of wavelengths (λ) 376–387 nm and 381–392 nm. The optimum reduction temperature for photo-catalytic activity was within the range 350–400 °C and attributed to improved charge-separation. The materials that were subject to carbo-thermal reduction at temperatures of 350 °C and 400 °C exhibited electrical conductivities over one hundred times higher compared to the non-reduced rutile. The results highlight that sub-oxide phases form an important alternative approach to doping with other elements to improve the photo-catalytic performance of TiO_2_. Such materials are important for applications such as self-cleaning where particles can be incorporated into surface coatings.

## 1. Introduction

Following the discovery of water photo-splitting by Fujishima and Honda in 1972 [1] the properties of titanium dioxide have received much attention [2]. Research has focused on a diverse range of applications including wastewater treatment, reduction of atmospheric air pollutants and neutralisation of bacteria, fungus and other pathogens [3,4,5,6]. In addition to these, TiO_2_ is also a key component in many self-cleaning technologies. Titanium dioxide is the natural oxide of Ti^4+^ (TiO_2_) with the three main crystallographic structures being anatase, brookite and rutile [7,8,9,10]. Rutile is the most common polymorph of titania on Earth due to its thermodynamic stability and low molecular volume and much of the titanium dioxide in metamorphic and igneous rocks has a rutile crystal structure. While the optical band gap of anatase (3.2 eV) is higher than rutile (3.0 eV) [11]. The photo-catalytic reactions promoted by rutile remain of interest in both the gas and aqueous phase due to its abundance and widespread use. Aqueous based photo-catalysis studies, under UV light, include those focusing on the degradation of organic dyes and neutralisation of pathogens [2,12].

Following the initial absorption of UV irradiation by the semiconducting rutile phase, an electron is promoted from its valence band to the conduction band over a femtosecond timescale [13]. This charge separation produces a reactive hole (h_vb_^+^) and associated electron (e_cb_^−^), this is shown in Equation (1). Both of these species may diffuse to the surface of the semiconductor and participate in a photo-catalytic reaction, provided they do not recombine forming an electron-hole pair. If the species are present on the surface, they can react with absorbed reduction and oxidation (red-ox) molecules leading to the formation of free radicals [1,14,15,16]. Equations (2)–(6) show typical reactions for H_2_O and O_2_ and those reactions which involve the formation of hydroxyl radicals and superoxide anions are the most important for pollution degradation, such as volatile organic compounds (VOCs) and NO_x_ via oxidation [6].

(1) $$TiO_{2}+h\upsilon \to h_{vb}^{+}+e_{cb}^{-}$$

(2) $$h_{vb}^{+}+H_{2}O\to {}^{•}OH+H^{+}$$

(3) $$e_{cb}^{-}+H^{+}\to {}^{•}H$$

(4) $$e_{cb}^{-}+O_{2}\to O_{2}^{•-}$$

(5) $$h_{vb}^{+}+O_{2}^{•-}\to {}^{1}O_{2}$$

(6) $$H^{+}+O_{2}^{•-}\to HO_{2}^{•}$$

Previous research on the surface of jet spray formed TiO_2_ suggested reduction of CO_2_ via reaction with an electron from the conduction band. The final reaction product formed following a number of reaction steps was proposed to be ethenolate [17]. In a different study the photocatalytic reduction of CO_2_ in aqueous solution on surface-fluorinated anatase TiO_2_ nanosheets with exposed {001} facets were investigated. Reduction of CO_2_ was proposed to proceed via a mechanism involving generation of hydrogen radicals and carbon radicals where CO_2_^−^ was formed extending the lifetime of photogenerated electron−hole pairs [18]. CO_2_ photo reduction has also been proposed to consume electrons and hydrogen radicals as shown in Equations (7)–(12) [19,20,21,22].

(7) $$CO_{2}+e_{cb}^{-}\to {}^{•}CO_{2}^{-}$$

(8) $${}^{•}CO_{2}^{-}+8{}^{•}H+h_{vb}^{+}\to CH_{4}+2H_{2}O$$

(9) $${}^{•}CO_{2}^{-}+6{}^{•}H+h_{vb}^{+}\to CH_{3}OH+H_{2}O$$

(10) $${}^{•}CO_{2}^{-}+2{}^{•}H+h_{vb}^{+}\to HCOOH$$

(11) $${}^{•}CO_{2}^{-}+4{}^{•}H+h_{vb}^{+}\to HCHO+H_{2}O$$

(12) $$2{}^{•}CO_{2}^{-}+12{}^{•}H+2h_{vb}^{+}\to C_{2}H_{5}OH+3H_{2}O$$

Halmann reported that one of the potential reasons why CO_2_ reduction may occur in a less extensive percentage is the reoxidation of CO_2_^−^ by hydroxyl radical [23,24]. Other studies suggested the mechanism involved the anchoring of CO_2_ on the TiO_2_ surface [25,26]. 

In order to improve the photo-catalytic performance, research has explored a variety of approaches to shift the absorbance spectra towards the higher wavelengths of visible light. These approaches include (i) doping of the TiO_2_, (ii) deforming its crystal lattice and (iii) tuning its bandgap [27,28,29,30]. Adding red-ox species, such as alcohols or ions such as Ag^+^, also slows down the recombination of electrons and holes thereby reducing the carrier losses. An alternative strategy is to modify the electronic transfer properties of the material and involves improving the diffusion of charge to the material surface by increasing the bulk electrical conductivity. 

In addition to the photocatalytic properties of TiO_2_ another important phenomena observed is that of superhydrophillic behavior. When self-cleaning mechanisms are considered, on very flat surfaces, such as glass, the superhydrophillic properties of TiO_2_ under UV irradiation can promote the formation of a thin water film rather than droplets, due to the reduced contact angle. This promotes the water film to run off the glass surface readily during which dirt particles are carried with it due to surface tension effects. However, this process is less likely to occur on rough or porous construction materials such as cement renders, lime renders, or the surface of concrete. On these rougher surfaced materials photocatalytic particles are added to the wet-mix. Following application of the cement, particles are left exposed on the surface following hardening or setting which impart self-cleaning properties due to the chemical degradation of dirt particles at the surface [31]. It is noteworthy that this mechanism is effective for organic contaminants containing bonds which can be attacked by the free radicals formed on the TiO_2_ surface under light irradiation. The process efficiency is linked to the photocatalytic properties of the material. 

In this paper we explore the approach of increasing the charge transfer towards the surface by reducing Ti^4+^ to Ti^3+^ to form sub-oxides and increase its electrical conductivity [32,33]. A variety of methods can be used to prepare titanium sub-oxides including hydrogenation [34,35], thermal treatment under an inert atmosphere [36], or by solvo-thermal methods employing TiCl_3_ and TiF_4_ [37]. This research investigates how vacancies in the rutile lattice of carbo-thermally reduced titania influence the phase structure, band gap, electrical conductivity and solid-gas photo-catalytic decomposition of nitrogen dioxide (NO_2_) and carbon dioxide (CO_2_) greenhouse gases. Photo-catalytic performance was evaluated under radiation of two different wavelength ranges, 376–387 and 381–392 nm, emitted from ultraviolet LED’s. The effectiveness of these sub-oxide materials to decompose NO_2_ is related to the free radicals formed on the surface. As it is these same radicals which react with other organic contaminates on the surface this can also reflect the self-cleaning potential. 

## 2. Materials and Methods 

Materials were formed from commercial TiO_2_ rutile powder (0.3 mm mean particle size, 99.5% pure, Pi-kem, UK) which was processed by the addition of 2.5% wt of an organic binder (polyethylene glycol-PEG 8000, Sigma Aldrich, Gillingham, UK). By adding 1% *v*/*w* of distilled water to the powder, a slurry was formed and ball milled in a Capco machine for 24 h at 120 rpm [38,39]. Ball milling reduced the particle size and thoroughly mixed the organic binder with the ceramic powder preventing the formation of agglomerates. The slurry was subsequently dried and the resulting powder sieved through a 45 μm mesh [40]. TiO_2_ specimens were formed by dry cold pressing at a pressure of 250 MPa (20 kN over an area of 100 mm^2^). The resulting green body was then pressure-less sintered in a furnace (LTF, Lenton, Nottingham, UK) at 1300 °C in air for 90 min with an initial dwell at 400 °C for 2 h to decompose the binder. This firing regime was previously optimised to maximise the density (98.65 ± 0.48% of theoretical) and control grain growth (5–10 µm) [41]. To introduce Ti^3+^ defects into the structure of the TiO_2_ a carbo-thermal reduction was employed, Equation (13) [42].

(13) $$nTiO_{2}+\frac{1}{2}C\to Ti_{n}O_{2n-1}+\frac{1}{2}CO_{2}$$

Carbo-thermal reduction of the sintered titania was performed in a tube furnace (LTF, Lenton, Nottingham, UK), under a constant flow of argon to prevent re-oxidation. A micro-environment was created by embedding the specimens in carbon powder consisting of needle shaped particles of mean diameter 3.55 µm and length 105 µm. The reduction treatment started with a heating ramp of 150 °C/h followed by a reduction stage of 24 h. A range of reduction temperatures was employed to vary the degree of reduction; these temperatures included 350, 400, 450, 600 and 1300 °C; a reduction temperature of 1300 °C is typical for the manufacture of highly reduced Magnéli phases [36,43]. Once the reduction was complete, samples were cooled to room temperature with a ramp of 150 °C/h. Further details describing the methodology for producing the sub oxide phases are available in PhD thesis [44]. 

The density of the samples was determined using the Archimedes method as described in the standard BS EN623:2.26 [45]. Theoretical densities (Dt) for TiO_2_ and TiO_n_ were assumed to be 4.26 g/cm^3^ [46] and 4.30 g/cm^3^ respectively.

X-ray diffraction analysis (XRD) was used to determine the crystalline phase composition of the specimens. XRD was carried out using a BRUKER D8 ADVANCE X-ray diffractometer (Bruker Ltd, Coventry, UK) with CuK_α_ radiation (1.5406 Å), 2θ range between 20° and 140° with a step size of 0.016° 2θ and a virtual time-per-step of 200 s. The microstructure of the specimens’ surface was evaluated using a JEOL-JSM64802V scanning electron microscope (SEM, Jeol Ltd., Herts, UK) with an accelerator voltage of 15 kV and a spot-size of 30 nm. Specimens were coated with a 10 nm layer of chromium to prevent surface charging. Rietveld refinement method was used for a fine study of oxygen occupancy. 

X-ray photoelectron spectroscopy (XPS, SPECS GmbH, Berlin, Germany) was used to measure the surface elemental composition. The system was equipped with a hemispherical electro energy analyser (SPECS Phoibos 150), a delay-line detector and a monochromatic AlK_α_ (1486.74 eV) X-ray source. High resolution spectra were recorded at normal emission take-off angle and with a pass-energy of 20 eV. The binding energies were corrected by referencing C-C to 284.8 eV. The optical bandgap energy of the materials was determined using a Shimadzu UV 3100 diffuse reflectance spectrometer (Shimadzu, Kyoto, Japan), employing the Kubelka-Munk function [34] (Equation (14)) to convert the reflectance, R, into the absorption coefficient, *F*(*R*).

(14) $$F\left(R\right)={\left(1-R\right)}^{2}/2R$$

Combining the Kubelka-Munk function with the Tauc’s plot provides the absorption energy which corresponds to the bandgap, *E_g_*, calculated from Equation (15),

(15) $${\left(h\upsilon \times F\left(R\right)\right)}^{2}=A\times \left(h\upsilon -E_{g}\right)$$

In addition to the bandgap, the diffuse reflectance spectrogram at 650 nm was used to assign the intervalence charge transfer (IVCT) transition between Ti^3+^ and Ti^4+^, where *h* is Planck’s constant, υ is frequency of vibration and ‘*A*’ is a proportionally constant. The bandgap was calculated from absorption spectra obtained at room temperature using a PerkinElmer 750 S UV/Vis Spectrometer with a 60 mm integrating sphere in a wavelength range between 240 nm and 800 nm [33]. 

The effect of level of reduction and Ti^3+^ on the ac conductivity within the specimen bulk was determined by impedance spectroscopy. Electrical properties of stoichiometric titanium dioxide and sub-oxide species were characterized using a Solartron 1260 Impedance Analyser (Solartron Analytical, Farnborough, UK), with a Solartron 1296 Dielectric Interface. Measurements were taken at frequencies from 1 to 10^5^ Hz with a voltage perturbation of 0.1 V_rms_. 

**Figure 1:**
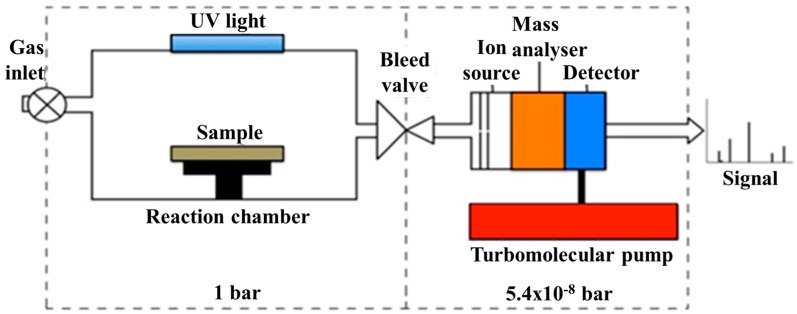
Schematic of the instrument for photo-catalytic activity.

The photo-catalytic activity of the TiO_n_ specimens was evaluated by following the decomposition of CO_2_ and NO_2_ under UV illumination over a duration of 150 min in a reaction chamber coupled to an electron ionization mass spectrometer, as shown in Figure 1. 190 ppm of NO_2_, 6% of air balanced N_2_ was introduced into the chamber followed by vacuum cycles up to 10^−3^ mbar to ensure the chamber filled with the gas mixture and was not diluted by the gas previously in the chamber. Under these conditions three separate experiments were conducted, in the dark and under UV LED’s emitting light of wavelength (λ) 376–387 nm and 381–392 nm. Experiments in the dark provided a comparison for absorption of gas onto the surface. Variations were shown to be within the background noise of the measurements. Further details can be found in the PhD thesis, Photocatalytic coatings in the built environment [47]. 

Data from the mass spectrometer was processed using a multiplicative approach using argon as an inert internal standard to avoid time-deviation errors [10]. The fractional change of inert argon during the experiment is defined as α, Equation (16), where the superscript ‘max’ is the maximum value of its partial pressure for the data series; ‘min’ is the minimum value of its partial pressure for the data series and ‘*i*’ is the partial pressure value for a given time during the experiment. A similar approach to data is then used relating to the relative change in molecule X of interest (e.g., CO_2_ or NO_2_); defined as ‘*ε_x_*’ in Equation (17). This treatment normalises the data to a value between 0 and 1. The fractional change of ‘X’ considering the changes of Ar with time by the area of the sample in the Ar partial pressure scale is then defined by Equation (18).

(16) $$\alpha =\frac{\left(Ar^{max}-Ar^{i}\right)}{\left(Ar^{max}-Ar^{min}\right)}$$

(17) $$\varepsilon_{X}=\frac{\left(X^{max}-X^{i}\right)}{\left(X^{max}-X^{min}\right)}$$

(18) $${}_{N}^{i}X=(\varepsilon_{x}-\alpha )/Area$$

After 150 min, the fractional change value of the component ‘X’ for the reaction under UV was subtracted from the fractional change value of ‘X’ from the control experiment with no specimen in the chamber. This approach was then applied to determine the changes of CO_2_ and NO_2_ with time under dark and illumination conditions.

## 3. Results and Discussion

### 3.1. X-ray Characterisation and Electron Microscopy

**Figure 2:**
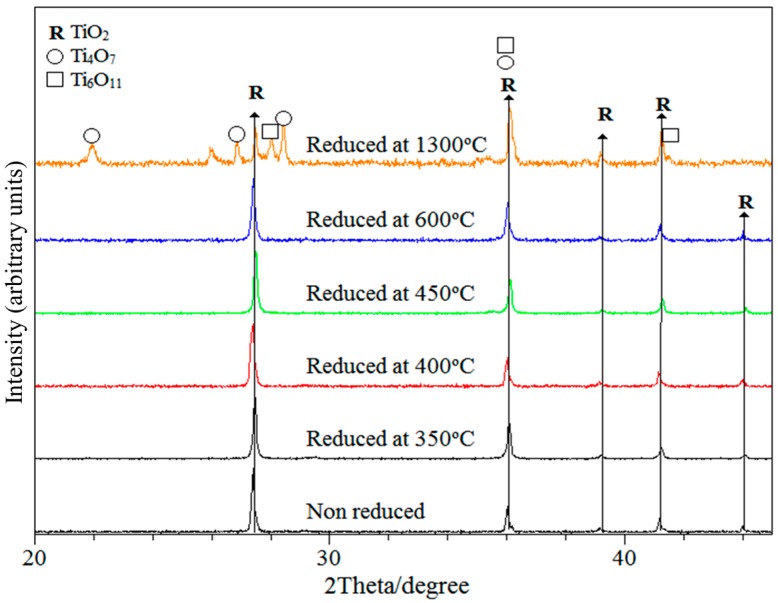
XRD diffraction patterns of non-reduced rutile and TiO_n_ carbo-thermally reduced from 350 °C to 1300 °C.

Crystalline phases were identified from the X-ray patterns. The XRD diffractogram, shown in Figure 2 indicates that rutile was the main crystallographic phase, even for the specimen reduced at 1300 °C where Magnéli phases (Ti_4_O_7_, Ti_6_O_11_) were present. The presence of secondary phases (i.e., sub-stoichiometric TiO_2−x_) in specimens reduced within the temperature range 350–600 °C was not detected; if present, these phases, related to defects within the TiO_2_ lattice, are therefore below the detection limit of the XRD system (approximately 5%). Nevertheless, the presence of defects in reduced specimens, particularly Ti^3+^, is confirmed by the changes in electrical conductivity measured by impedance spectroscopy, to be discussed in Section 3.3. 

**Figure 3:**
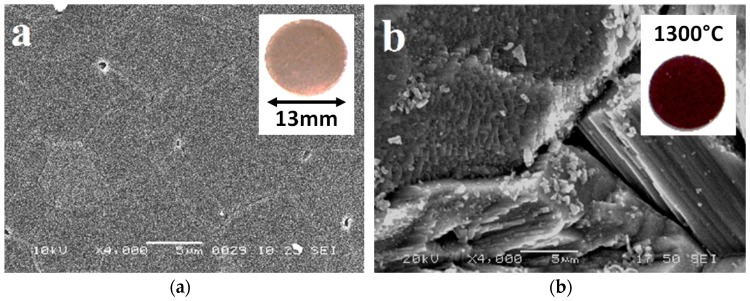
SEM images at same scale of (**a**) non-reduced TiO_2_; (**b**) TiO_2_ carbo-thermally reduced at 1300 °C. Inset shows colour change of material.

Changes in specimen microstructure as a result of the reduction temperature was examined by scanning electron microscopy, revealing no significant changes in structure from the non-reduced TiO_2_ and specimens reduced at temperatures up to 600 °C. Figure 3a,b show the surface morphologies of the non-reduced TiO_2_ (theoretical density ~98.6%) and Magnéli phase based material reduced at 1300 °C (theoretical density ~96.7%) respectively. Due to the increased conductivity and diffusion processes during the reduction of TiO_2_, there is significant grain growth when the material is reduced at this high temperature. The carbon-thermal reduction process leads to a gradual darkening on the materials as the reduction temperature increases; see insets Figure 3a,b. 

**Figure 4:**
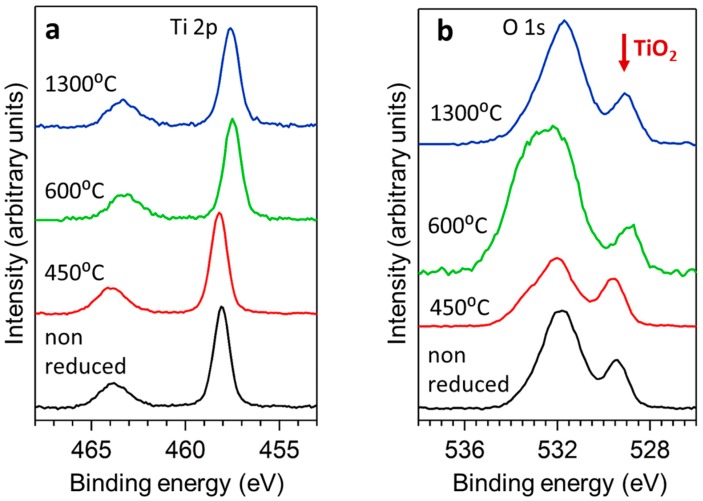
X-ray photoelectron spectra in the Ti 2p (**a**) and O 1s (**b**) regions of non-reduced rutile and TiO_n_ carbon-thermally reduced from 450 °C to 1300 °C.

Figure 4a,b show the X-ray photoelectron spectra (XPS) obtained from the samples in the Ti 2p and O 1s regions. In the case of the non-reduced samples, Ti 2p_3/2_ presents a sharp component centered at a binding energy (BE) of 458.1 eV. This value shifted 0.5 eV towards lower binding energies for the samples prepared at higher temperatures 600 °C and 1300 °C of Figure 4a. Complementary, this shift was also detected in the O 1s core level as it is shown in Figure 4b, where the small component on the right of the spectra is ascribed to the oxygen atoms in the TiO_2_ structure. These results are in good agreement with previous works in which the shift to lower BEs were ascribed to a reduction process [48].

### 3.2. UV-Visible Diffuse Reflectance Spectroscopy 

Changes in band gap with reduction conditions, were identified by the application of the Kubelka-Munk model, however this was not applicable for the sample reduced at 1300 °C containing significant conducting Magnéli phase as it was no longer a semiconductor; see impedance data in Section 3.3. Only small changes in the band gap were observed with the maximum corresponding to the specimen reduced at 400 °C, see Table 1.

**Figure 5:**
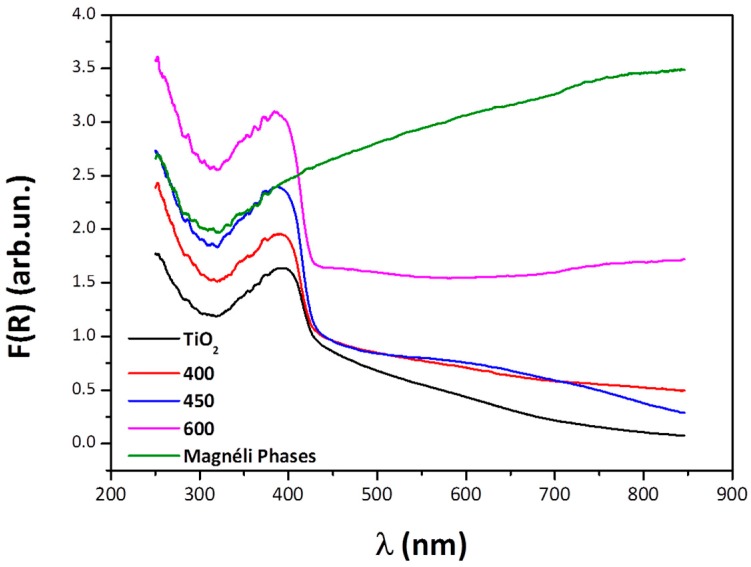
Diffuse reflectance spectra for rutile and reduced samples.

The diffuse reflectance spectra in Figure 5 revealed an absorption band at 650 nm for the samples reduced at 450 °C which can be assigned to the intervalence charge transfer transition Ti^3+^ to Ti^4+^.

### 3.3. Electrical Characterization

**Figure 6:**
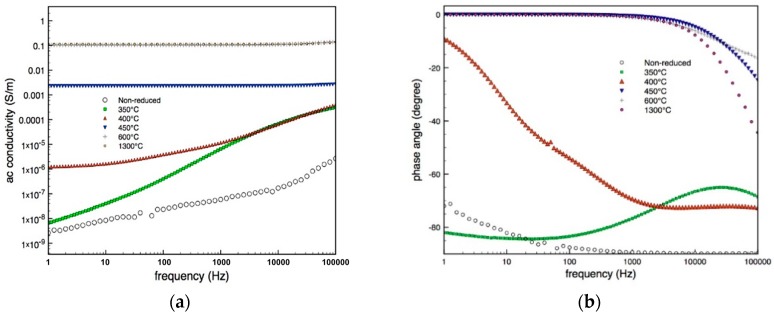

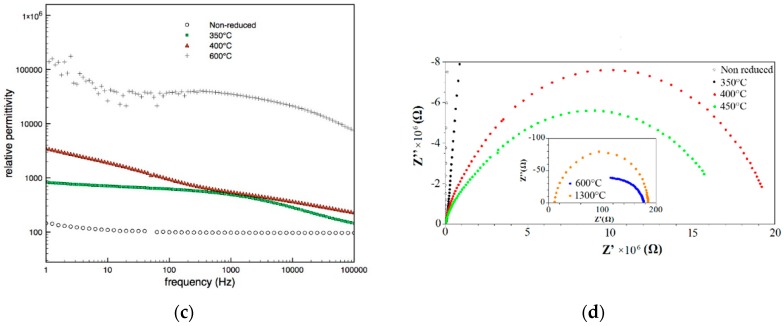
(**a**) AC conductivity (**b**) phase angle (**c**) permittivity, (**d**) complex plane plot of real impedance (Z’) vs imaginary impedance (Z’’) for TiO_n_ specimens.

Impedance spectroscopy was performed to identify correlations between the ac conductivity and the photo-activity of the specimens. Figure 6a shows that the ac conductivity of the non-reduced rutile phase rises almost linearly with frequency and in this case the material is behaving predominately as a dielectric and the phase angle approaches −90° since current in a capacitor (dielectric) leads the voltage; see Figure 6b. For the carbo-thermally reduced materials there is an increase in the low frequency conductivity with increasing reduction temperature, see Figure 6a. Materials reduced at 350–400 °C exhibit the ‘universal dielectric response’ (UDR) whereby at low frequency the bulk ac conductivity is frequency independent, σ_dc_, while at higher frequencies the ac conductivity increases following a power law behavior [49,50]. The higher electrical conductivity with increasing reduction temperature is a clear indication that by increasing the reduction temperature, a larger number of defects is introduced within the rutile lattice. It can therefore be concluded that, although the presence of Ti^3+^ could not be detected by XRD, a reduction temperature of 350 °C is sufficient to form Ti^3+^ states. 

In specimens reduced at 450 °C to 1300 °C the defects are the dominant phase and the conductivity response is frequency independent and the materials no longer exhibit the UDR, Figure 6a. This behaviour is much like that of a resistor with the phase angle approaching 0° and current and voltage are in phase, Figure 6b. Figure 6c shows the relative permittivity of selected materials as a function of frequency. The non-reduced rutile phase exhibits a relatively frequency independent permittivity, typical of a dielectric. The materials reduced at 350 °C and 400 °C show a significantly enhanced relative permittivity (in excess of 103) and strong frequency dependence. Materials reduced at 450–1300 °C showed excessive noise in the permittivity data due to the high conductivity; e.g., see data for 600 °C where the relative permittivity was 10^4^–10^5^; such as enhancement in permittivity, and frequency dependence has been shown to originate from conductivity in the material.

Changes in the conductivity are also shown in Figure 6d, illustrating the frequency dependence of the complex impedance, again indicating the transition from capacitive behaviour to restive behaviour as the material is carbo-thermally reduced with a corresponding increase in electrical conductivity; this can be seen as a decrease in the real part of impedance (*Z’*) at low frequencies (right hand side intercept of the *Z’*-axis).

### 3.4. Photo-Catalytic Performance

**Figure 7:**
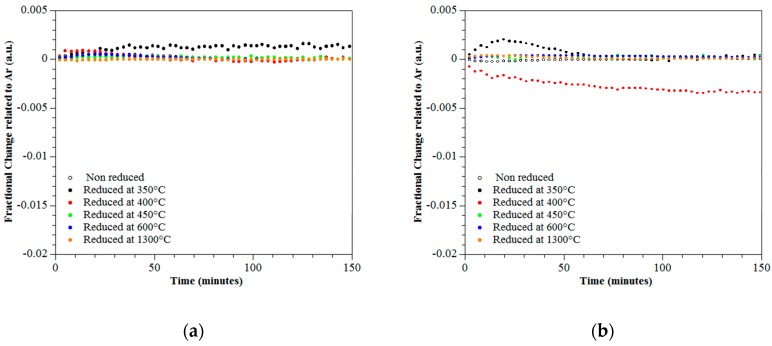
Fractional change of CO_2_ over 150 min under UV of wavelengths (**a**) 376–387 nm and (**b**) 381–392 nm.

**Figure 8:**
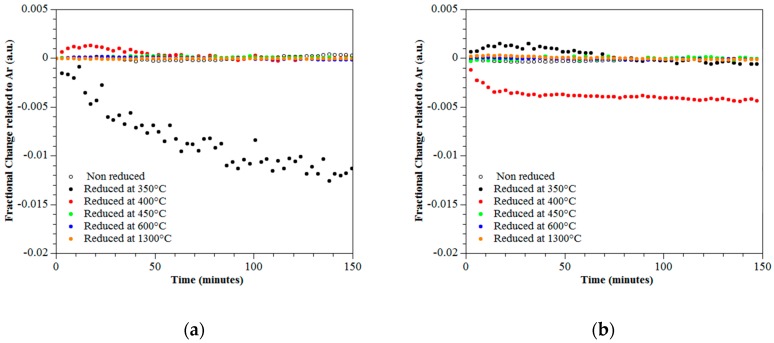
Fractional change of NO_2_ over 150 min under UV wavelengths of (**a**) 376–387 nm and (**b**) 381–392 nm.

The degradation of CO_2_ and NO_2_ was monitored over 150 min through the fractional change relative to Ar for rutile and titanium sub-oxides. The sub-oxides were produced by reduction of rutile at temperatures ranging from 300 °C to 1300 °C. Two independent experiments were conducted using irradiation from LED’s with wavelengths ranging from 376–387 nm and 381–392 nm. Figure 7a,b present results showing the fractional change of CO_2_ under wavelengths of 376–387 nm and 381–392 nm respectively. Figure 8a,b present results showing the fractional change of NO_2_ under wavelengths of 376–387 nm and 381–392 nm respectively. The results show an increase of photo-activity for both NO_2_ and CO_2_ when subjected to irradiation at wavelengths of 381–392 nm for the titanium sub-oxide sample reduced at 400 °C; see Figure 7b and Figure 8b. The maximum degradation of NO_2_ was observed for the titanium sub-oxide reduced at 350 °C when irradiated with a wavelength of 376–387 nm; see Figure 8a. 

Based on these results it is thought that when the TiO_2_ was not reduced the defects introduced within the rutile lattice are insufficient to induce a significant change in the conductivity of TiO_2_ and improve charge-separation. The low frequency ac conductivity for the sample reduced at 350 °C increased by a factor of 10^1^, compared to non-reduced rutile, see Figure 6c, and up to 10^3^ for the sample reduced at 400 °C, leading to enhanced photo-activity.

This increase in the conductivity, due to the presence of Ti^3+^ in the bulk, can improve the efficiency of the electron-hole separation and stabilisation of the charge distribution. As a result, the photo-activity of the material increased. When the material was subjected to a greater degree of reduction, i.e., at reduction temperatures above 450 °C, a much higher concentration of defects is produced which leads to localisation of defects, promoting recombination of charge carriers and therefore reducing the photo-activity. Tailoring the degree of reduction therefore offers a route to balance these effects to produce improved photo-catalytic materials. 

## 4. Conclusions

This paper has demonstrated that solid-gas phase solar photo-catalysis of rutile based materials can be enhanced by production of TiO_n_ (1 < n < 2) phases prepared by carbo-thermal reduction under an inert atmosphere. Materials were formed by carbo-thermal reduction of rutile at temperatures from 350 °C to 1300 °C and characterised in detail. The optimum temperature range to enhance photo-catalytic properties was 350 °C to 400 °C and demonstrates that the photo-catalytic performance of TiO_2_ can be improved by the introduction of defects into the lattice by a reduction process. 

The enhancement in solid-gas phase photo-activity for the degradation of CO_2_ and NO_2_ was attributed to the increase in the electrical conductivity; this leads to an improved separation of photo-generated charge carriers. Subjecting rutile to reduction temperatures 450 °C and above lead to a significant increase in conductivity and created localized titanium sub-oxide species leading to the promotion of recombination of charge carriers and therefore does not lead to enhanced photo-activity. Impedance spectroscopy has proven to be the most suitable technique to measure titanium sub-oxide dislocations due to its sensitivity. This strategy constitutes an important alternative to doping TiO_2_ with other elements to modify the band gap and thus photo-catalytic performance for applications such as air purification.

## Figures and Tables

**Table 1:** Optical band gap of TiO_n_ specimens.

Specimen-Reduction Temp	Band Gap (eV)	Rietveld Refinement for Oxygen Vacancies
Rutile TiO_2_	3.03	1
TiO_n_-350 °C	3.04	1
TiO_n_-400 °C	3.06	1
TiO_n_-450 °C	3.04	1
TiO_n_-600 °C	3.05	0.95
TiO_n_-1300 °C	Overlapped bands	-
